# The Development of a Syringe-Based Insulin Applicator Using a Biodesign-Based Methodology

**DOI:** 10.3390/biomimetics11060394

**Published:** 2026-06-03

**Authors:** Alejandro A. Salinas-Aguilar, Sebastian Arriaga-Marin, Carlos A. Perez-Ramirez, Ignacio Cervantes-Gutierrez, Irving A. Cruz-Albarran, Andres Emilio Hurtado-Perez, Manuel Toledano-Ayala

**Affiliations:** Tequexquite, Centro de Investigación y Desarrollo Tecnológico para la Accesibilidad e Innovación Social, Facultad de Ingeniería, Universidad Autónoma de Querétaro, Campus Aeropuerto, Carretera a Chichimequillas S/N, Ejido Bolaños, Querétaro 76140, Mexico; alejandro.salinas@uaq.mx (A.A.S.-A.); sebastianarriagamarin@gmail.com (S.A.-M.); carlos.perez@uaq.mx (C.A.P.-R.); ignacio.cervantes@uaq.mx (I.C.-G.); irving.cruz@uaq.mx (I.A.C.-A.)

**Keywords:** biodesign methodology, insuline applicator, industrial design

## Abstract

Effective diabetes management heavily relies on appropriate insulin administration, which strongly depends on the correct administration strategy. In this sense, insulin administration plays a fundamental role, as its use depends on the patient’s clinical condition and diabetes type. Traditional syringe-based methods require proper training to ensure that insulin is successfully delivered into the subcutaneous tissue, where it can be absorbed and metabolized; however, it is desirable to develop an insulin applicator that does not require training for its appropriate use. Aiming to provide support solutions that help patients to develop a correct administration technique, a biodesign-based methodology, coupled with biomimetic concepts, is employed to design a device that assists the user in creating a stable skin fold and guiding needle orientation during injection without requiring exhaustive training for device usage. A three-step approach is employed for the design, where computational fluid dynamics (CFD) and finite element analysis (FEA) methods are employed to ensure that the device produces a laminar insulin flow and the device strength is tested. It should be pointed out both methods are required since complications produced by sudden flows must be avoided, with CFD allowing assessment of the device mechanical properties in terms of the device strength. Initial functional evaluation indicates that the proposed approach does not require extensive training or complex operational procedures, facilitating its integration into everyday use. The device design is validated from the results obtained for the CFD analysis, as no turbulent flow is produced, whereas the FEA indicates that the geometrical form can handle the stresses produced by the folding generation without generating excessive deformations. Moreover, an infrared thermography analysis is also carried out to find out if the folding force generation is located in the zone of interest, the results of which indicate that the device operates in the desired physical zone.

## 1. Introduction

According to studies published by the Pan American Health Organization (PAHO) [1,2], type-2 diabetes mellitus prevalence is estimated to be about 13% of the adult population, with Mexico being among the countries that are the most affected [3,4,5], with the reported prevalence there about 14.4% of the adult population [3,6,7,8]. These data are reinforced by the rate reported by the International Diabetes Federation [5], where for 2024, the prevalence is 16.4%. These numbers indicate an alarming increase with potentially catastrophic consequences for societal well-being, as increasing numbers of adults may develop associated life-threatening illness that has both significant time and economic implications.

Diabetes is defined as a chronic and degenerative disease that is produced by insufficient insulin production [9]. It is well known that inadequate insulin levels affect blood glucose level, which, in the long term, can cause structural damage to nerves and blood vessels [9]. In this sense, the introduction of insulin has transformed diabetes management, since its usage is associated with disease chronification [10,11]. This scenario has produced new challenges, such as the risk of hypoglycemia and cardiovascular complications [12,13,14,15], highlighting the importance of ensuring its proper and safe administration, with particular emphasis on vulnerable populations [12].

**Figure 1:**
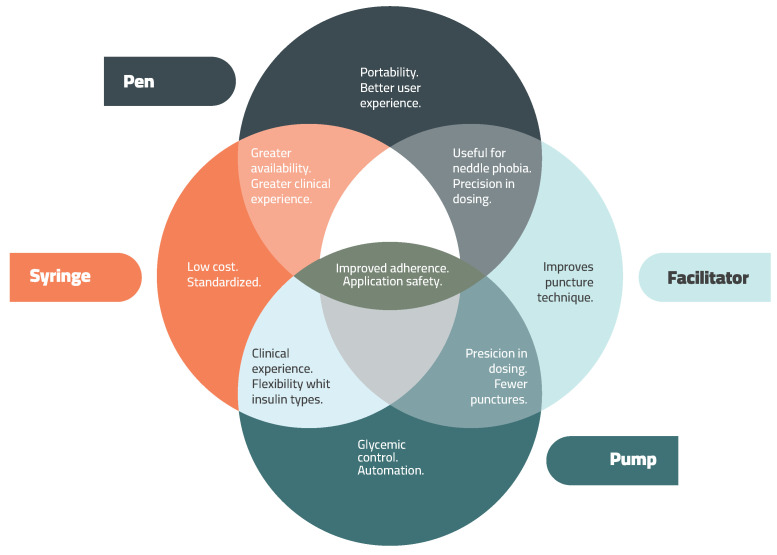
Comparative advantages of insulin delivery devices based on clinical and user-centered criteria.

Different methods for insulin administration that patients with diabetes employ have been reported. Their usage depends on factors such as the diabetes type, lifestyle, economic status, tolerance to injections, as well as the physiological response to treatment [16,17,18]. The most commonly employed methods are syringes, insulin pens, sensor-free and sensor-integrated insuline pumps, as well as hybrid systems [19]. Chaya et al. [12] note that patients face both adaptive and emotional challenges that require the provison of information to enable an informed choice. Results presented by Shashank et al. [19], Kesavadev et al. [20], and Aideen et al. [21] illustrate the benefits and limitations of these devices. A graphical resume of the various devices’ advantages is presented in Figure 1. It should be noted that the central zone in the figure represents the desired design target, that is, optimal adherence usage and safe application procedure.

**Figure 2:**
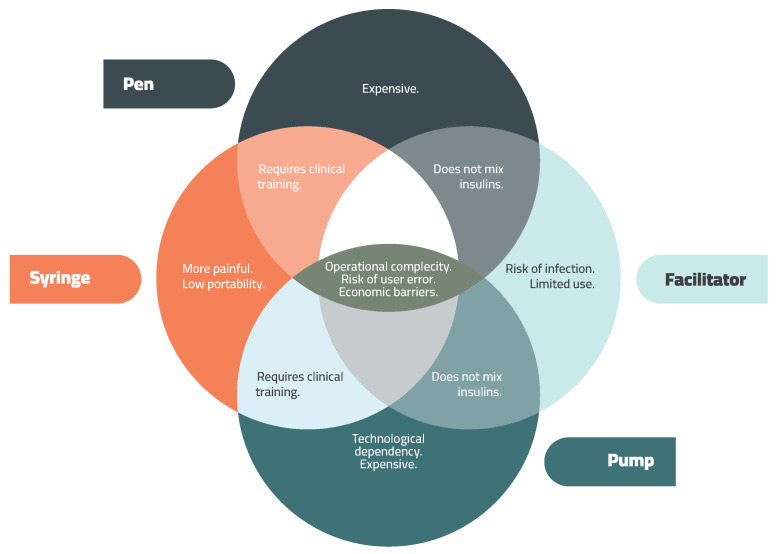
Comparative disadvantages of insulin delivery devices based on clinical and user-centered criteria.

The devices’ disadvantages are summarized in Figure 2. The central zone denotes the worst-scenario, that is, a combination of limitations, rather than the disadvantages that all devices simultaneously have.

From Figure 1 and Figure 2, it can be concluded that the design approach should prioritize device adherence and usage, as well as ensuring its safe use, while avoiding high operational complexity that can lead to user errors, or economic barriers that may hinder device access or usage adherence. The term adherence is defined as the degree to which a person’s behavior corresponds with the recommendations agreed with a healthcare professional [22,23]. Effective communication between patients and healthcare professionals is of paramount importance, as it supports proper device use, and collaborative assessment of adherence challenges [24]. A user-centered approach can ensure provision of a device with the aforementioned features.

The adherence rate for utilization of insulin pens in terms of therapy continuity is higher than that of other devices [22]; yet, they involve greater operational complexity, as patients must calculate, record, and recall their insulin doses. While hybrid options, such as smart pens, have emerged to reduce this burden, they still require further exploration and evaluation [25,26]. Insulin pumps have demonstrated appropriate therapeutic adherence but the operational complexity of appropriate device use is still an issue [26]. Injection facilitators such as the I-Port Advance have shown improvement in the adherence rate by reducing skin punctures, thus increasing acceptance among pediatric populations [27,28]. In a similar way, mechanical autoinjectors have demonstrated greater adoption rates as they minimize technical variability during application, ensuring correct angles and depths are used. Consequently, the pain and anxiety produced during the procedure are reduced. These factors collectively enhance adherence, although their higher cost compared to traditional syringes remains a limitation [29]. This aspect is of paramount importance in middle- and low-income countries, as insulin applicators are considered ’out-of-pocket’ items that patients must pay for [30].

Considering the abovementioned scenarios, traditional syringes remain the most employed option [29]. The main challenge regarding adherence lies in proper application, i.e., clinical training, as injection technique is the main factor that influences device adherence [24,31]. Therefore, an approach that ensures correct application while maintaining the use of traditional syringes is still needed.

Biodesign methodology provides a framework for user-centered medical innovation, whose guiding principle is the identification of real clinical opportunities. This approach integrates end user needs from the earliest stages, considering physiological, ergonomic, emotional, and contextual factors that influence technology adoption [32]. Recent studies have shown that this approach fosters an empathetic understanding of both patients and healthcare professionals, resulting in devices that are safer, more intuitive to use, and more sustainable [33,34]. The methodology is structured into three iterative phases: (1) identification, (2) invention, and (3) implementation [35]. It should also be noted that the development of medical devices can also benefit from biomimetic approaches, where forms, structures, and functional mechanisms are developed by a process of natural evolution and translated into engineering solutions. Biomimetics aims to replicate efficient natural strategies that optimize interaction, adaptability, and mechanical performance under specific constraints [36]. In biomedical applications, this approach has found particular relevance in systems requiring controlled deformation, surface adaptation, and safe interaction with biological tissues, especially where skin-contact and soft-material interfaces are involved [37,38,39,40,41].

This paper adopts a biodesign methodology combined with a biomimetic approach as the guiding framework for developing a mechanical facilitator for insulin administration. The proposed design aims to enhance precision and comfort during injection while maintaining the simplicity, low cost, and autonomy inherent to the use of traditional syringes. Through a structured process of needs identification, user-centered ideation, and iterative clinical validation, the developed solution can help to strengthen therapeutic adherence and improve user experience for insulin delivery, thus aligning technological innovation with the real-life clinical and emotional requirements of individuals living with diabetes. Specifically, the device design draws inspiration from natural shapes, such as curvature-shaped forms, that have sufficient flexibility to generate controlled interactions on soft surfaces, which are often found on human tissues, such as those generated by bone cells [42]. Using this approach, a compliant geometry capable of producing a stable skin fold and facilitating consistent needle orientation during injection can be developed. By integrating these biomimetic insights with a user-centered biodesign methodology [32,33,34,35], the proposed device aligns functional efficiency with simplicity, adaptability, and safe interaction with the human body [43].

## 2. The Biodesign-Based Methodology

**Figure 3:**
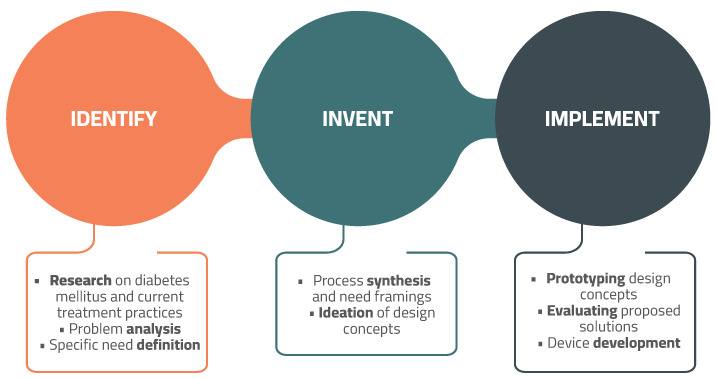
Biodesign process employed for the development of the insulin injection facilitator.

Figure 3 graphically depicts the biodesign-based methodology as well as the required activities for developing the device.

**Figure 4:**
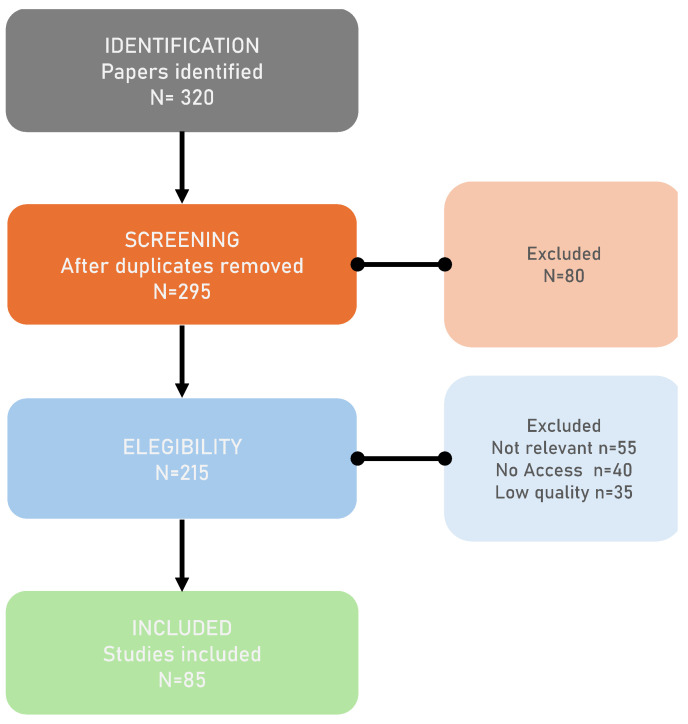
Literature selection process flow diagram.

A structured literature selection process was undertaken to identify relevant studies that can support the proposed device development. This research was performed using three scientific databases: PubMed, Scopus, and Google Scholar. The topic searches were related to insulin administration techniques, device design, and user-related factors. Keywords such as *insulin injection technique*, *subcutaneous injection*, *insulin delivery devices*, and *patient adherence* were used. The selection process followed a PRISMA-based approach, where identification, screening, eligibility, and inclusion stages were the main guiding factors. These are summarized in Figure 4. Initially, records were identified through database search and screened based on title and abstract relevance. Subsequently, full-text articles were assessed considering their relevance to injection technique, mechanical assistance, and clinical applicability. Studies were excluded if they were not directly related to the research objective or had restricted access. This process resulted in a final set of studies that informed both the clinical and engineering aspects of the proposed device.

### 2.1. Identify

The first development step focuses on identifying the insulin administration pathways in Mexico, where a state-of-the-art investigation was conducted on type-2 diabetes mellitus, as this type of diabetes has the highest prevalence [3]. From this investigation, it was concluded that an unresolved clinical need regarding insulin administration using traditional syringes existed. Here, adherence performance and personal barriers experienced by users were the main guiding variables.

From the abovementioned investigation, traditional syringes emerged as the most used administration method, where the main detected anomalies include the use of expired insulin, syringe reuse, inaccurate dosing, and intramuscular injection due to the needle not being tilted at 45°. These findings show that clinical problems are not related to the device itself, but to the injection technique, where a lack of education, economic barriers, limited user-centered design, and an absence of standardization in the procedure are noted as the factors that contribute the most to the injection technique. In summary, the core insights are as follows:

1. Users do not consciously integrate angle and depth as structured components of the injection process.
2. Dose visualization during injection is not intuitive, leading to inaccurate dosing.

To better assess these limitations, the key parameters involved in the injection process and their clinical implications are summarized in Table 1. This analysis allows the variables that directly affect the effectiveness of insulin administration to be identified, particularly those associated with user-dependent actions such as angle selection, depth control, and tissue interaction.

**Figure 5:**
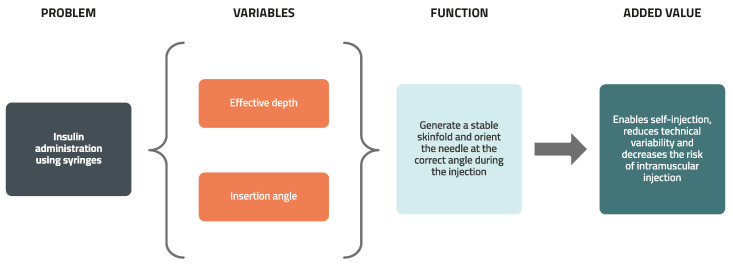
Biodesign-based necessity-solving process.

A problem identified is the miscalculation of both angle and depth during injection, which leads to incorrect subcutaneous administration and dependence on third parties to successfully perform the task. Figure 5 schematically represents this process.

In order to further contextualize the identified problem, it is necessary to analyze current insulin delivery assistance technologies and their functional capabilities. These systems have been developed to improve aspects such as dose tracking, monitoring, and automation; however, their contribution to the physical execution of the injection process remains limited. Table 2 presents an overview of commercially available insulin delivery assistance technologies, highlighting levels of automation, functionality, and key limitations.

From this comparison, it can be observed that most current devices focus on connectivity, digital integration, and automated dose management. However, they provide little to no mechanical assistance for controlling key variables such as insertion angle and depth during injection. This limitation reinforces the idea that the main gap in current technologies lies not in data management or monitoring, but in the lack of physical guidance during the injection process. This gap between the technologies’ capabilities and mechanical assistance represents the main opportunity addressed in this work.

### 2.2. Invent

Once the main function is defined, the next step consists in determining the preliminary specifications. At this stage, the design characteristics of conventional insulin syringes and needles are first considered, as they define the interaction between the user and the injection process. Table 3 summarizes the main geometric, mechanical, and material features that directly influence insulin delivery, highlighting how variables such as needle length, gauge, geometry, and mechanism require active user control during administration.

From this analysis, it can be observed that conventional syringe design inherently requires a high-level of user control, as multiple variables must be manually coordinated during injection. Bearing this in mind, the product is synthesized at a conceptual level, where the essential attributes required to generate a prototype are defined as:

1. Geometric form. An ergonomic geometry must be employed so the device can be held with one hand while providing a stable grip during application.
2. Dimensions. The device size must be compact and portable to facilitate its daily transport and storage.
3. Materials. The device must be manufactured using bio-compatible, hypoallergenic polymers that can be easily cleaned, while still providing adequate mechanical strength.
4. Operating principle. The mechanism must be fully mechanical and manually operated without integrated electronics, prioritizing simplicity, low cost, and a reduced learning curve.

**Figure 6:**
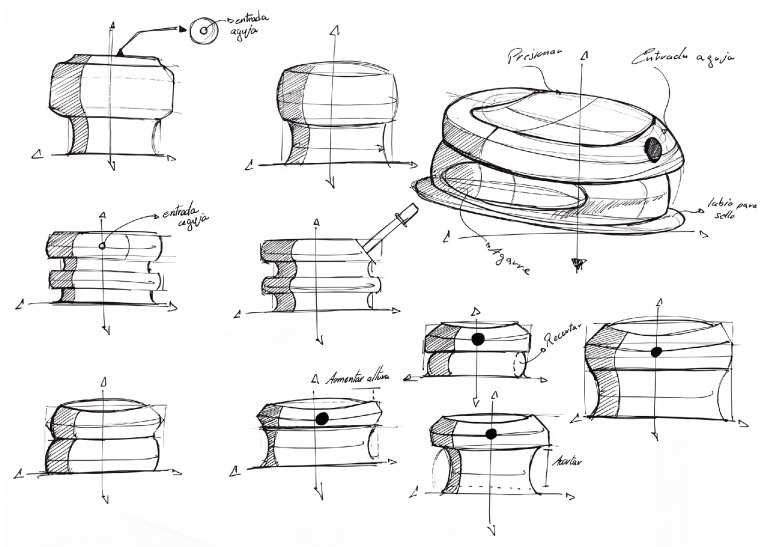
Ideation sketches exploring early form development for the insulin applicator.

Considering the abovedefined attributes, visual tools are employed to explore alternatives and iterations, enabling the construction of a visual narrative that guides the product language, the intended experience, and the message to be conveyed to the user. In this stage, the device conceptual pillars are set: facilitation, self-control, simplicity, and accessibility. In this sense, the device design can achieve the set goal, which is to provide an improved insulin-injection application process. Consequently, the lifestyle and daily context of people living with diabetes are conceptualized in order to set the design approach in real-life use scenarios, avoiding user exclusion as the central concern of product development. Finally, an aesthetic language is defined, emphasizing matt materials, soft tactile surfaces, and ergonomic forms, aiming to evoke perceptions of safety, cleanliness, and control. Once these three elements are defined, the design process can advance to the sketching phase, where the biomimetic concepts must also be considered. In this sense, curvature-based devices are known for generating folding surfaces [60]; hence, they must be considered as the initial device form. After these elements are defined, the design process can move to the sketching phase, where different configurations and geometric alternatives are explored. A graphical representation is presented in Figure 6.

### 2.3. Implement

**Figure 7:**
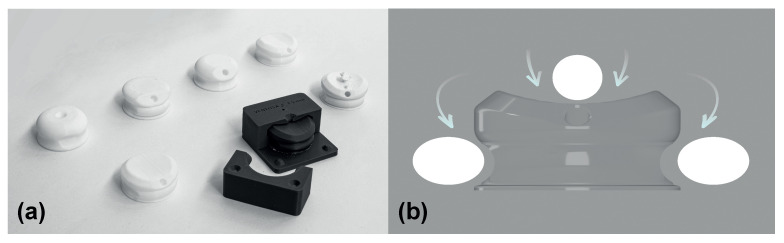
Development and operational principle of the syringe-based insulin applicator: (**a**) iterative prototypes and mold fabrication, (**b**) schematic representation of the device operation.

In the ideation stage, the development of functional prototypes using materials that could also be suitable for final manufacturing, such as silicone, polypropylene, and silicone-coated PTFE, is desired [37,38,61]. The prototype development process is shown in Figure 7a, where several iterations of the device and the mold fabrication employed to construct the silicone components are shown; the operational principle is also depicted in Figure 7b.

**Figure 8:**
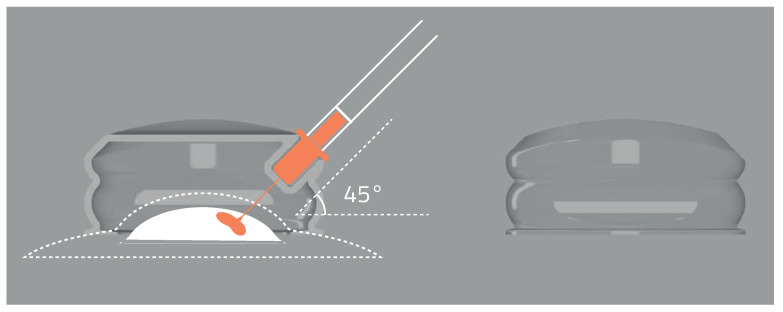
Schematic representation of the device operational principle.

The prototype is evaluated using four parameters: (1) functionality, (2) manufacturability, (3) usability, and (4) suction performance. These parameters are considered in order to preserve device adherence usage while maintaining the focus on user-centered design. The first parameter evaluates whether the device effectively generates a stable skin fold and orients the needle at approximately 45° for syringe insertion and subsequent subcutaneous delivery. This operational principle is illustrated in Figure 8.

Regarding usability, preliminary testing with different participants indicates that the device performs more effectively when manipulated using a three-finger interaction. In this configuration, the middle and thumb fingers stabilize the device central body while the index finger applies force on the upper silicone surface, allowing a predefined deformation during placement and activation. For suction performance, iterative testing indicates that adjustment device dimensions and contact area are necessary to increase surface adhesion, which, in consequence, will improve the generation of local negative pressure. These opportunities for improvement are reflected in Figure 7b, which highlights the design development modifications applied to device form and scale to enhance grip and suction performance.

The device’s manufacturability must also be considered; the device geometry requires the fabrication of silicone molds to reproduce the flexible structure that enables controlled deformation. Scalability and cost are considered by prioritizing injection-based manufacturing processes, which allow mass production; moreover, quality control is enhanced from mold-based fabrication, since standardized tolerances and repeatable inspection criteria can be applied to each produced unit.

Once the shape and geometry, user interaction with the device, interaction requirements in terms of adherence, affordability for the target population, and the clinical needs that the device must fulfill have been defined, material selection for the development is carried out. To this end, three requirements are considered [60]:

1. Flexibility and deformation capacity.
2. Appropriate interaction with human skin.
3. Stability under skin contact.

The material must be capable of deforming to achieve suction, generate the skin fold, and subsequently return to its original state. Based on these requirements, silicone is selected as the material due to its ability to fulfill the properties [37,38], as it has a high biocompatibility with human tissue [38,62,63]. This biocompatibility has been demonstrated under real clinical conditions, such as implant applications, where the use, deformation, and temperature variation are important factors that can compromise the device’s structural integrity. In these conditions, silicone shows non-toxicity and stability, maintaining its mechanical properties both in implantable applications and during cellular interaction [63,64]. Beyond its clinical use in implants, silicone has also demonstrated favorable properties when interacting directly with the skin, including uniform distribution, low irritation, and dermatological compatibility [65]. These features allow the material to perform its function, remain in contact with the skin, and recover its original shape [37].

In addition to these requirements, the use of silicone remains highly relevant in biomaterials, biomedical engineering, and clinical environments [66,67]. Furthermore, silicone can be easily sterilized and exhibits high durability, which supports its use in external devices intended for repeated use [68].

## 3. Methods and Materials

### 3.1. Employed Simulation Methods

The material employed in the device construction must have sufficient mechanical resistance for continuous use, generate suction on the skin to ensure fixation and fold formation, and finally, produce laminar flow during insulin injection. To test if the material and geometrical shape fulfill the abovedescribed tasks, three analyses are performed:

1. Finite element-based (FEA) static analysis. An evaluation of the device’s mechanical properties is undertaken to find out if the material and geometrical shape have sufficient strength for performing the suction.
2. Thermographic analysis. The suction and device attachment to the skin are tested using infrared thermography (IRT).
3. Computational fluid dynamics (CFD) analysis. The insulin flow analysis within the syringe is analyzed to determine if a laminar flow is generated.

The simulations are performed using SolidWorks 2022. Simulation modules are employed to evaluate structural integrity, stress distribution, and the ability to generate suction [69], as well as fluid dynamics. The model is defined as a continuous solid with elastomeric silicone-based properties [70], with a Poisson’s ratio of 0.49 representing quasi-impressible behavior [71]. Different device thickness configurations varying from 2.5 to 3.5 mm are analyzed to evaluate their mechanical response, as mechanical interaction and pressure distribution are critical design factors [72]. The models are analyzed using a curvature-based solid mesh with high-order quadratic elements, with sizes ranging from approximately 0.38 to 4.3 mm, ensuring mesh quality. Boundary conditions are defined based on a realistic scenario, including a fixed constraint at the base of the device representing skin contact, the application of a 15 N force on the upper surface corresponding to the force exerted by a functional adult [73], and a distributed pressure of 98 kPa representing the generated suction.

Additionally, for an insulin delivery device, performance does not depend solely on a structural design, but also on the device–skin interface, where contact mechanics play a fundamental role [39]. This performance can be evaluated through mechanical interaction by incorporating negative pressure, contact, and deformation effects to ensure safety and functionality [74]. The skin response to the suction generated by the device is evaluated using infrared thermography (IRT), as this technique allows the early detection of tissue alterations caused by pressure, since it detects temperature changes associated with variations in blood pressure [75,76]. This makes IRT a suitable tool for analyzing localized mechanical interactions, such as suction or pressure generated by a medical device in contact with the skin.

The experimental protocol is designed under a pre–post measurement scheme, where a thermographic image is recorded before and after device application, evaluating two different thickness configurations (2.5 and 3.5 mm), aiming to analyze the thickness influence on the skin thermal response. The thermographic analysis considers both physiological variability and external factors; therefore, variables such as skin tone, local mechanical interaction, and ambient conditions must be controlled to avoid influencing the results [77]. In this study, suction is generated through the device mechanical deformation on the skin surface for 10 s as part of the experimental protocol. These measurements ensure consistency between samples, as IRT demonstrated excellent reproducibility under controlled experimental conditions [78]. This interaction represents real-world device use, where negative pressure depends on material deformation and the force applied by the user. In the spatial analysis, regions of interest (ROIs) are defined corresponding to the center, edge, and periphery of the suction area to evaluate the thermal distribution associated with mechanical interaction. These values are analyzed using inferential statistics through a paired *t*-test to compare pre- and post-conditions within each device configuration.

Finally, to evaluate insulin behavior within the syringe, a computational fluid dynamics analysis is performed, allowing study of the interaction between the fluid and the delivery system geometry. These techniques can serve as effective tools for analyzing and optimizing insulin injection systems, as they integrate mechanical, fluid, and biological phenomena within a single framework [79]. The model is developed in SolidWorks Flow Simulation under an internal flow approach, considering the geometry of a U-100 syringe. The computational domain is defined with approximate dimensions of 0.057 m for the main axes and 0.005 m in the transverse axis to ensure proper representation of the system volume. A structured mesh with approximately 25,000 fluid cells is used, allowing capture of the main flow characteristics within the conduit without compromising numerical stability and convergence based on the SolidWorks Flow Simulation goal-based criteria [80]. The fluid is modeled as water, considering that most insulin formulations present viscosity values close to that of water at room temperature, enabling a valid approximation for flow analysis [81]. Additionally, recent studies report that the viscosity of insulin analog ranges from 1.064 to 1.146 mPa · s, reinforcing the validity of this simplification in the CFD model [82]. This consideration is consistent with approaches where the physicochemical properties of the fluid are fundamental in determining injection behavior and delivery efficiency [83].

Furthermore, boundary conditions are defined using a mass flow inlet of $1.6\times 10^{-5}$$\frac{\mathrm{kg}}{s}$, equivalent to a volumetric flow rate on the order of 0.0016 $\frac{\mathrm{mL}}{s}$ for an aqueous solution, a magnitude consistent with low-volume manual subcutaneous injection and clinically derivable estimates for U-100 syringe [84]. In this regard, an ambient pressure condition of 101.325 kPa is set, according to the definition of static pressure for outlet conditions in internal CFD flows, representing a manual injection scenario. Additional conditions include a temperature of 293.2 K, zero initial velocity, and a laminar flow regime assumption so that the system behavior depends solely on the imposed conditions. All these considerations are set to ensure that the physical parameters and boundary conditions are representative and comparable to real conditions, thereby guaranteeing the validity of the CFD model [85].

### 3.2. Employed Materials and Manufacturing Techniques

Once the simulations are carried out, it is necessary to generate the device prototypes to generate the test models. For the first stage, a morphological validation is carried out, where a thermoplastic polyurethane (TPU) filament-based model is generated using a QIDI Q2 3D printer (Shenzhen Qidi Technology Co., Ltd., Shenzhen, China). It should be pointed out that this prototype allows determination of the device’s ergonomic interaction using a practical approach.

**Figure 9:**
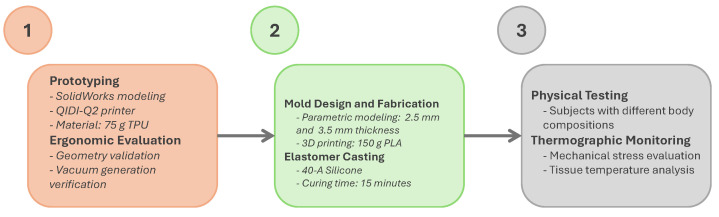
Proposed device manufacturing process and experimental validation stages employed during development.

After the first evaluation is completed, the manufacturing process can print functional prototypes with mechanical properties closer to those of the final device. For this purpose, polylactic acid (PLA) molds are designed in SolidWorks and fabricated using additive manufacturing. These molds are later used for casting silicone 40-A resin-based prototypes, which are cured for 20 min. Consequently, the device’s structural integrity can be assessed using a practical approach. Two prototypes are produced, where their thickness is varied: 2.5 mm and 3.5 mm. Both configurations are designed for skin fold formation and localized suction. Their validation is achieved using IRT to determine the interaction and attachment with the skin under operational conditions. The overall manufacturing workflow employed during the development of the proposed device is depicted in Figure 9.

## 4. Results

### 4.1. Identify

The identification stage indicated that the primary barrier in syringe-based insulin administration is the variability associated with the injection technique. The analysis of current practices shows that users often fail to consciously integrate key parameters such as insertion angle and effective depth during injection, which may lead to incorrect subcutaneous administration or dependence on third parties to perform the procedure [31].

In consequence, these variables are processed to generate a functional objective involving generating a stable skin fold, thus enabling the needle orientation during injection. This conceptual translation of the clinical need into design variables provided the foundation for the subsequent development stages.

**Figure 10:**
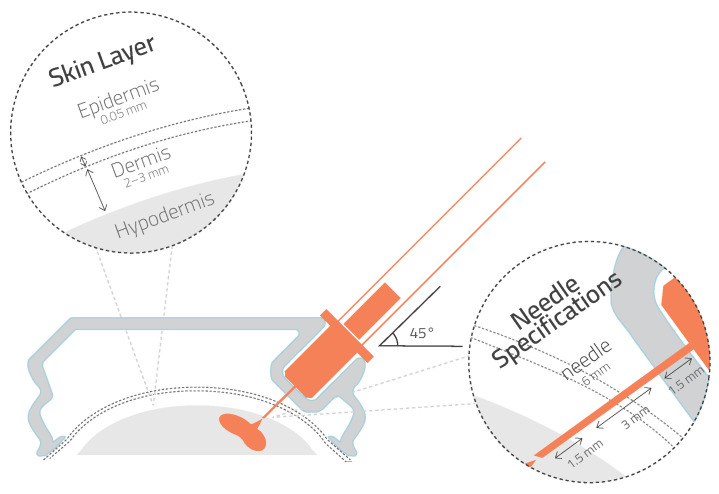
Developed geometrical shape for generating the appropriate skin fold.

A stable skin fold is produced when the device generates a tissue movement, so the insulin is injected in the hypodermis rather than other places [44]. In this sense, it must be considered that the depth of both the dermis and hypodermis is between 2 and 3 mm [46], whereas the complete subcutaneous tissue depth depends on the amount of fatty tissue [46]. Considering these factors, an exhaustive study is carried out to determine the geometric shapes based on curvature that can generate the required tissue movement when a 6 mm needle (standard size) is employed. Figure 10 illustrates the resulting device. It can be seen that even when mechanical movement is generated, the needle insertion angle remains constant around 45°.

Once the clinical and geometric requirements, namely, the 45° insertion angle, controlled depth, and skin fold generated, as illustrated in Figure 10, are defined, a CFD analysis is performed to evaluate whether insulin delivery using a U-100 syringe remains feasible under these conditions. This analysis ensures that the proposed mechanical interaction does not negatively affect the fluid behavior during injection [79].

**Figure 11:**
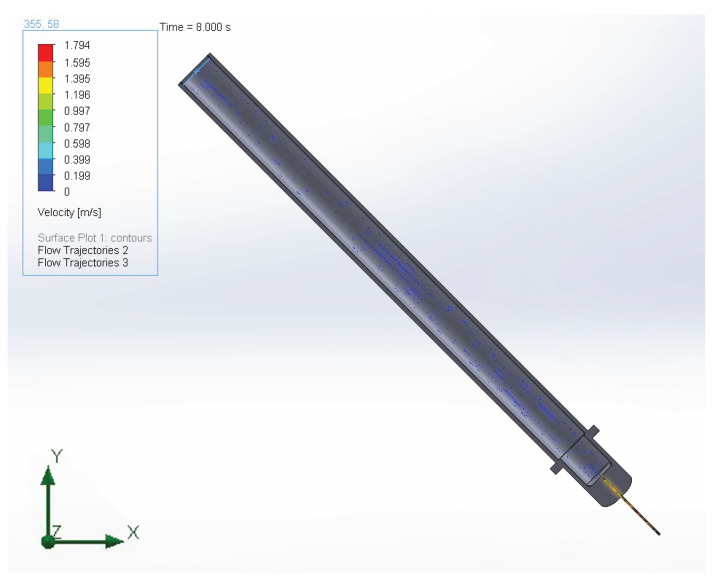
Velocity distribution and flow trajectories inside syringe obtained from CFD.

The CFD simulation allows evaluation of key fluid parameters within the syringe, including density, pressure, velocity, shear stress, and relative pressure, in order to characterize the internal flow behavior during injection. From the CFD simulation results obtained for the proposed device, the main fluid parameters are summarized in Table 4. The fluid density remained constant at approximately 997.56 $\frac{\mathrm{kg}}{m^{3}}$, confirming that the model fluid behaves consistently with an incompressible aqueous solution, which is a valid approximation for insulin formulation due to its low viscosity and water-like behavior [81,82]. The pressure values ranged from 101.32456 to 127.14982 kPa, indicating the presence of a pressure gradient driving the flow through the syringe and needle, which is expected for a pressure-driven injection system [83]. The velocity distribution reached a maximum value of 1.794 $\frac{m}{s}$, with localized increases along the flow path, particularly near geometrical restrictions, as expected in confined internal flows [83]. Despite these variations, the flow remains stable and well defined, as illustrated in Figure 11, where the velocity contours and flow trajectories show a continuous and organized flow pattern along the syringe.

The shear stress values ranged from 0 to 90.88 Pa, remaining within acceptable limits for fluid transport injection systems, suggesting that the flow conditions do not induce excessive mechanical stress on the device, which is critical to preserve the physicochemical integrity of insulin [82,83]. The relative pressure varied between −0.44 and 25.82482 kPa, reflecting localized pressure changes associated with the internal geometry and confirming the presence of a controlled flow regime [83]. Overall, the flow remained predominantly laminar, which is consistent with low flow rates and small characteristic dimensions typical of subcutaneous injection systems. This laminar behavior ensures a stable and predictable flow, contributing to improved dosing control and reducing the risk of flow-induced disturbances [83,85].

### 4.2. Invent

**Figure 12:**
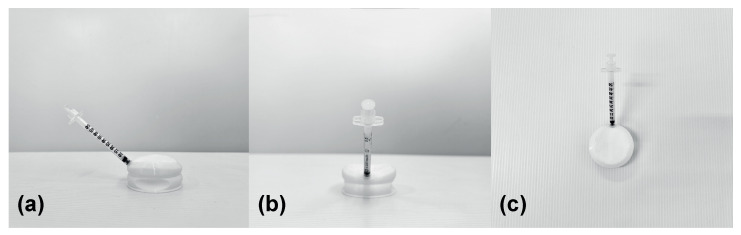
Device operation from (**a**) horizontal, (**b**) frontal, and (**c**) vertical view.

Based on the previously identified variables, the invention stage focuses on translating the functional requirements into conceptual design attributes. The design exploration process considers ergonomic interaction, portability, and mechanical simplicity as the main product attributes, since these factors are critical in the usability and effectiveness of medical devices [45,47]. Through iterative conceptual exploration and sketch-based ideation, several geometric configurations are evaluated to determine how the device could interact with the user’s hand while generating a stable skin fold during placement, taking into account the importance of controlled tissue deformation in subcutaneous injection procedures [44,45,58]. This process led to the definition of a flexible applicator concept capable of producing localized deformation when external pressure is applied. The design approach emphasized a fully mechanical system that could guide the injection process without requiring electronic components or complex operation, aligning with approaches that prioritize simplicity and accessibility in medical devices. The device operation is depicted in Figure 12.

Once the conceptual design and its interaction with the insulin flow are set, the analysis is extended to evaluate the device mechanical behavior under operational conditions. In this context, finite element analysis (FEA) is employed to assess stress distribution, deformation, and structural response. This technique is widely used to validate mechanical performance and pressure distribution in skin-contact medical devices [72,74].

**Figure 13:**
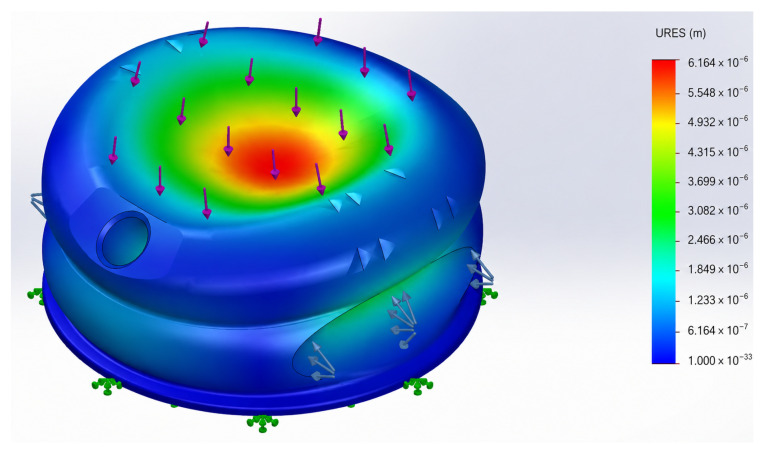
Displacement distribution (URES) obtained from finite element analysis, showing localized deformation in the central region.

Figure 13 shows the displacement distribution (URES), where deformation is concentrated in the central region of the device. This localized behavior is essential for generating the required skin fold, while the peripheral regions remain mechanically stable. Such controlled deformation is critical in subcutaneous injection procedures, where tissue displacement directly influences the correct delivery of insulin into the hypodermis [44,46]. To quantitatively evaluate the mechanical response, the main simulation results for both configurations are summarized in Table 5. The reported parameters describe the structural behavior of the device under the applied loading conditions obtained from the FEA simulation. Von Mises stresses represent the internal stress distribution generated within the structure during deformation, whereas displacement corresponds to the magnitude of structural movement produced by the applied load. Similarly, the minimum and maximum displacement values describe the structural deformation induced by mechanical loading, whereas the strain-related behavior reflects the material compliance during deformation; on the other hand, the reaction force represents the mechanical response generated at the constrained regions of the device, while the applied force and pressure correspond to the external loading conditions defined during the simulation. Finally, the number of elements and nodes describes the mesh generation employed for the numerical solution of the finite element model.

As shown in Table 5, the 2.5 mm configuration shows a significantly higher maximum displacement compared to the 3.5 mm configuration, indicating greater flexibility and higher capacity to deform under the same loading conditions. This increases deformability and directly supports the generation of a skin fold, which is a critical requirement for proper subcutaneous insulin delivery [44]. In terms of stress distribution, the thinner configuration also shows higher von Mises stress values, approximately doubling the ones observed in the thicker configuration. This behavior is consistent with the reduced structural stiffness associated with thinner geometries and reflects the trade-off between flexibility and mechanical resistance in elastomeric systems [39]. Similarly, strain values follow the same trend, with the 2.5 mm configuration resulting in higher deformation levels when compared to the 3.5 mm model, confirming that thickness directly influences the device mechanical compliance [39,74]. Finally, the reaction forces remain consistent across both configurations, confirming that boundary conditions and applied load are equivalent, allowing for a direct comparison between geometries.

### 4.3. Implement

**Figure 14:**
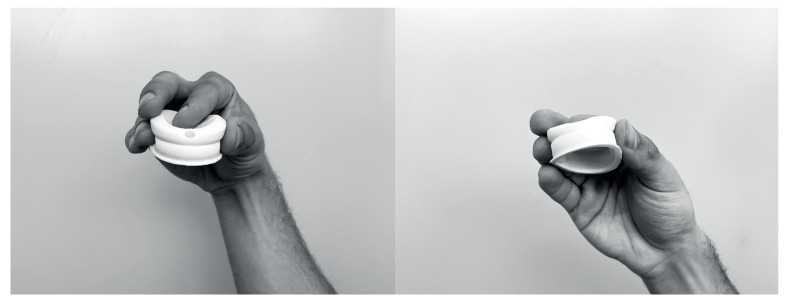
Three-finger interaction with silicone applicator prototype. The device is stabilized using the thumb and middle finger while the index finger applies pressure on the upper surface, producing a controlled deformation of the silicone body that enables stable positioning on the skin before injection.

The implementation stage focused on translating the conceptual design into a functional prototype using flexible silicone components. Several iterations of the device were developed to refine geometry, grip configuration, and surface contact features. The resulting prototype enables a controlled interaction between the user and the applicator flexible body. As shown in Figure 14, the device can be manipulated using a three-finger configuration in which the thumb and middle fingers stabilize the device while the index finger applies downward pressure on the upper silicone surface. This interaction produces a controlled deformation of the flexible structure, allowing the device to adapt to the curvature of the skin while maintaining stability during placement.

**Figure 15:**
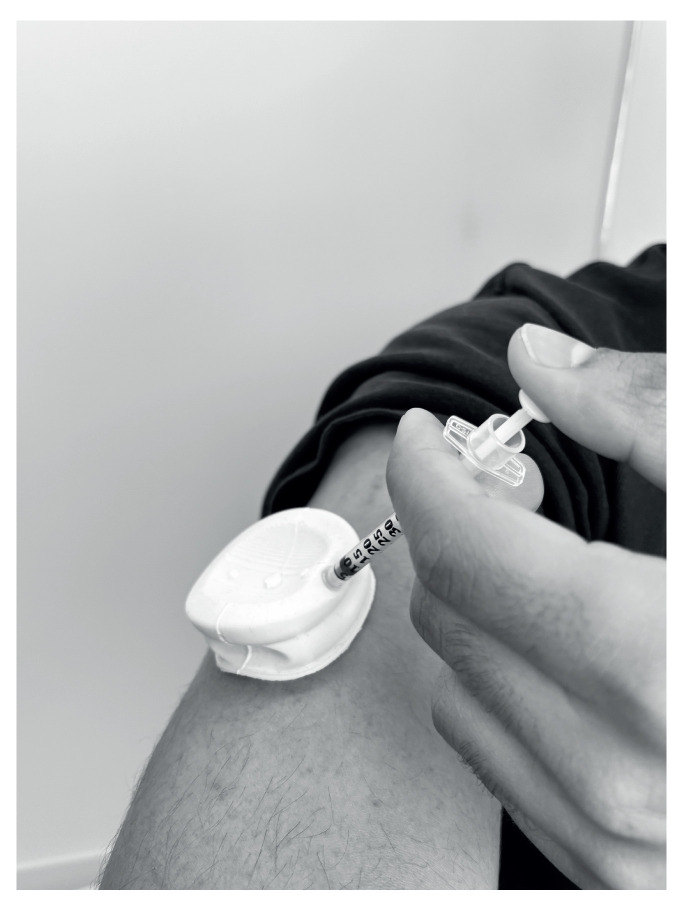
Application scenario of the silicone insulin applicator prototype during insulin administration.

The silicone body deformation generates a localized skin fold and increases the contact area between the device and the skin surface. This behavior stabilizes the injection interface and prepares the application area for syringe insertion. The application scenario is depicted in Figure 15, where the device is positioned on the arm before injection. In this configuration, the device maintains the skin fold while allowing the syringe to be positioned through the guide interface while preserving compatibility with standard insulin syringes.

**Figure 16:**
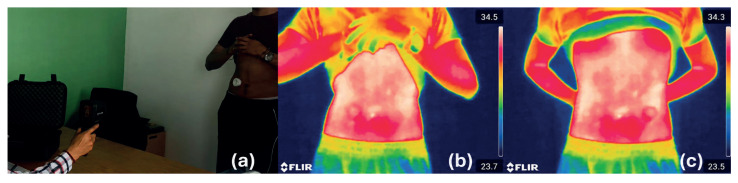
Infrared thermography evaluation of skin response under suction conditions: (**a**) experimental setup, (**b**) thermal distribution after application of the 2.5 mm device on the right side, and (**c**) thermal distribution after application of the 3.5 mm device on the left side, showing localized temperature variations associated with mechanical interaction.

To evaluate the skin physiological response under suction conditions, IRT is employed as it allows the temperature assessment variations associated with localized mechanical interaction. This technique has been widely validated as a non-invasive method for detecting early tissue responses related to pressure and perfusion changes [75,76]. Figure 16 presents the thermographic evaluation, including the experimental setup (a) and the thermal distribution after device application (b,c), where localized temperature changes can be observed in the region of interest.

Quantitatively, the descriptive analysis shows a decrease in mean temperature after device application for both configurations. For the 2.5 mm configuration, it decreases from 32.90 °C to 32.51 °C, while for the 3.5 mm configuration, it decreases from 33.07 °C to 32.70 °C. These changes suggest a localized physiological response associated with the mechanical interaction between the device and the skin.

The statistical analysis confirmed that these differences are significant. The paired *t*-test yielded t=6.063 (p=0.0005) for the 2.5 mm configuration and t=5.788 (p=0.0007) for the 3.5 mm configuration, indicating a statistically significant reduction in temperature after device application in both cases. The Shapiro–Wilk test confirmed normality in all datasets (p>0.05), supporting the use of parametric statistical methods. These results indicate that the device induces a consistent and measurable thermal response, associated with localized changes in skin interaction; such behavior is consistent with previous studies where thermographic variations reflect pressure-induced physiological changes in tissue [75,77].

It should be observed that the thermal response confirms that the device is capable of generating sufficient mechanical force on the skin to produce a measurable physiological effect, thereby validating the device suction functionality as intended. This is consistent with reported ranges of adhesion and mechanical interaction required for a stable skin–device interface [86]. Furthermore, the reproducibility and consistency of the thermal patterns support the reliability of the measurement approach [78].

**Figure 17:**
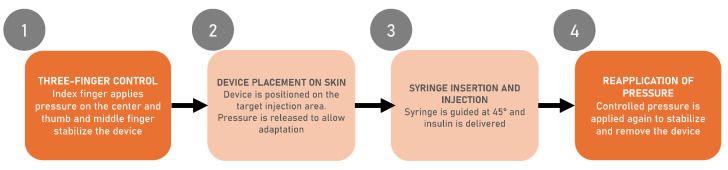
Device operation flow diagram.

Once the proposed device has been physically designed as well as its main properties have been defined, a flow diagram that can indicate how the device operates is highly desirable. Figure 17 presents the main steps for employing the device. From the figure, it can be seen that four steps are required, which are described as follows: (1) the device is placed on the skin area where the insulin will be injected; then, (2) using a three-finger configuration, where the index one presses the device center, the skin fold is generated; after that, (3) the device has a syringe port where it is guided with a 45-degree port to introduce the syringe with the insulin; then, the insulin is injected. Finally, (4) by repeating the collocation procedure, the device can be safely removed.

## 5. Discussion

The results obtained through the identification, invention, and implementation stages show how the biodesign-based methodology enables the systematic translation of a clinical need into a functional design concept. As illustrated in Figure 1 and Figure 2, current insulin delivery technologies have unique advantages and disadvantages. While advanced systems such as insulin pens and pumps provide dosing precision and/or automation, they often involve higher economic cost and operational complexity [19,21,81,87,88]. Conversely, traditional syringes remain widely accessible and standardized but strongly depend on proper injection technique and user training. This contrast highlights an important research area [22,24,25]. As shown in Figure 1, the ideal design objective lies in achieving both safe application and improved adherence while maintaining simplicity and accessibility; however, as summarized in Figure 2, existing devices rarely combine these characteristics simultaneously, since improvements in dosing precision or automation are typically associated with increased complexity or cost [19,21,81].

In this context, the biodesign framework provides a structured methodology for identifying and translating unmet clinical needs into design opportunities. As depicted in Figure 3, the methodology organizes the innovation process into three iterative stages: identification, invention, and implementation. This structure allows the problem to be explored from a clinical and user-centered perspective before proposing technological solutions [32,33,34,35].

During the identification phase, the analysis of insulin administration practices revealed that the principal barrier associated with syringe use is the variability introduced by the injection technique [31,45,89]. The schematic representation presented in Figure 5 shows how this clinical need can be translated into key design variables, specifically, insertion angle and effective depth, which directly influence correct subcutaneous administration [44,45,46]. Based on this functional framing, the invention stage focused on defining design attributes capable of addressing these variables through a user-centered approach [32,35,47]. Instead of modifying the syringe itself, the design process explores the development of an auxiliary device capable of stabilizing the injection interface and assisting the user during the preparation stage of the injection procedure [28,31,58]. Finally, the implementation stage demonstrated that a flexible silicone applicator can reproduce the required functional behavior through controlled deformation and localized suction [37,38,63,64,65]. The resulting device enables the formation of a stable skin fold while maintaining compatibility with standard insulin syringes, thereby addressing the identified need without introducing additional technological complexity [44,45].

From a broader perspective, as well as the economic restraints that low-income patients face in middle-industrialized countries, the preferred choice must be to complement traditional application methods, i.e., traditional syringes, by assisting the injection technique [30,87]. In this sense, the results illustrated how the biodesign methodology can guide the development of practical solutions by linking clinical observations, functional variables, and prototyping with a user-centered design process. A qualitative comparison between the proposed applicator and other insulin delivery approaches is presented in Table 6, highlighting how the device maintains the accessibility of traditional syringes while supporting key aspects of the injection technique.

Besides the conceptual and qualitative analysis, data obtained from Table 1, Table 2 and Table 3, coupled with the CFD, FEA, and IRT evaluations, provide a comprehensive validation of the proposed device. Table 1 confirms that variables such as insertion angle, depth, and skin fold are critical for achieving correct subcutaneous insulin delivery [44,45,46], while Table 2 highlights that current technologies mainly focus on monitoring and automation without providing mechanical guidance during injection [19,21,81]. In addition, Table 3 reinforces that traditional syringe use remains highly dependent on user technique, supporting the need for an auxiliary device that reduces variability in the application process [31,58,89].

From a functional perspective, the CFD results demonstrated that the proposed configuration maintains a stable and predominantly laminar flow, ensuring that insulin delivery is not compromised; thus, the dosing accuracy is preserved [81,82,83,85]. Similarly, the FEA results showed that both thickness configurations are capable of generating the deformation required to produce a stable skin fold, with the 2.5 mm configuration exhibiting greater mechanical compliance and adaptability to the skin interface [39,69,74]. Furthermore, the IRT analysis confirmed that both configurations generate a consistent physiological response associated with suction, validating the ability of the device to interact effectively with the skin under real conditions [75,76,77,78,86]. Considering that both thicknesses achieved functional performance, the 2.5 mm configuration should be used as the preferred design due to its lower material usage and reduced manufacturing cost, aligning to maintain accessibility and affordability without compromising performance [30,87,88].

## 6. Conclusions

This work presents the development of a syringe-based insulin applicator using the biodesign methodology coupled with biomimetic-inspired concepts as the guiding framework. Through the stages of identification, invention, and implementation, the development of a clinical need associated with injection technique variability into functional design variables and subsequently into a mechanical solution is carried out.

The results show how a user-centered innovation process can guide the development of devices aimed at supporting insulin administration while maintaining compatibility with traditional syringes. In particular, the proposed applicator enables the generation of skin folds and provides a stable interface for syringe positioning during the injection process. From a methodological perspective, this study demonstrates the usefulness of the biodesign methodology for structuring medical device development by linking clinical observations, design variables, and iterative prototyping. It should be pointed out that the device can be easily cleaned using traditional methods such as sterilization, which are well known and do not require any additional training or specialized cleaning products. The developed device operates using a 4-step procedure, where a three-finger movement allows the device to operate. To validate the device design, simulations regarding the insulin flow and the device, as well as the mechanical strength using CFD and FEA, are carried out. The obtained results affirm that the geometrical form and the material selection can fulfill the intended usage; moreover, an IRT analysis is also carried out to validate that the skin fold is generated as expected, as the skin temperature decreased its value when the fold is generated, which is an expected outcome, as the blood flow is instantaneously decreased.

Future work will focus on extensive usability evaluation and mechanical fine-tuning of the design to increase suction performance. Further, fatigue and remaining life tests should be undertaken to properly assess the product lifetime and possible failure rate, which are important aspects that must be considered for commercial production.

## Figures and Tables

**Table 1:** Overview of the main injection technique parameters and clinical impact.

Feature	Condition	Clinical Importance	Error Consequence	Proposed Device	References
Insertion Angle	45° or 90°	Determines correct subcutaneous delivery	Intramuscular injection or poor absorption	Guides 45° insertion	[44,45]
Needle Length	4 to 8 mm	Affects penetration depth	Over- or under-delivery	Compatible with standard needles	[31,45,46]
Skin Fold	Present and Absent	Ensures subcutaneous tissue targeting	Increased variability in delivery	Generates controlled skin fold	[44,45,46]
Injection Depth	Variable	Determines insulin absorption	Reduced efficacy or complications	Stabilizes interface	[31,44,45,46]
User Technique	Highly variable	Critical for correct administration	High variability in outcomes	Reduces user dependency	[31,44,47]
Dose Accuracy	Device dependent	Impacts glycemic control and adherence	Hypoglycemia or hyperglycemia	Indirectly improved via stability	[22,44]

**Table 2:** Overview of commercially available insulin delivery assistance technologies.

Device	Type	Main Functionality	Level of Automation	Injection Guidance	Main Disadvantages	References
InPen	Smart Pen	Dose tracking, Bluetooth connectivity	Medium	None	Requires user setup	[48,49,50]
Innovation Zed Cap	Smart Cap	Injection monitoring	Low–Medium	None	Limited integration	[51]
Bigfoot Unity	Smart Management System	Dose recommendation and CGM integration	High	None	High cost and ecosystem dependency	[52,53]
Tempo Smart Button	Smart Management System	Dose tracking and app integration	Medium	None	Requires compatible devices	[54]
NovoPen 6	Smart Pen	Memory, dose logging	Medium	None	Limited to tracking	[55]
NovoPen Echo Plus	Smart Pen	Memory and pediatric usability	Medium	None	Limited to tracking	[56]
YpsoMate	Autoinjector	Automated injection	High	Partial	High cost	[28,57]

**Table 3:** Design characteristics of insulin syringes and needles.

Feature	Values	Role	References
Needle length and gauge	4–8 mm/28 G–32 G	Penetration depth vs. flow resistance	[31,44,58]
Tip geometry and coating	Multi-bevel tip (3–5 facets), silicone-coated	Reduce insertion force and friction	[31,58]
Barrel volume and scale	0.3–1 mL/1–2 unit increments	Dose range and measurement precision	[44,59]
Plunger and dispenser	Manual	Control of fluid delivery accuracy	[59]
Materials (barrel/needle)	Polymer/stainless steel	Structural integrity and usability	[31]

**Table 4:** Summary of CFD parameters obtained for the proposed insulin delivery device.

Parameter	Minimum	Maximum
Density $\left(\frac{\mathrm{kg}}{m^{3}}\right)$	997.5	997.56
Pressure (Pa)	101,324.56	127,149.82
Relative pressure (Pa)	−0.44	25,824.82
Velocity magnitude ($\frac{m}{s}$)	0	1.794
Shear stress (Pa)	0	90.88

**Table 5:** Comparison of mechanical response between 2.5 mm and 3.5 mm thickness configurations.

Parameter	2.5 mm Thickness	3.5 mm Thickness
Max von Mises stress $\left(\frac{\mathrm{kgf}}{{\mathrm{cm}}^{2}}\right)$	14.06	7.52
Min von Mises stress $\left(\frac{\mathrm{kgf}}{{\mathrm{cm}}^{2}}\right)$	1.32 $\times \;10^{-2}$	
Max displacement (m)	6.03 $\times \;10^{-4}$	3.05 $\times \;10^{-4}$
Min displacement (m)	7.67 $\times \;10^{-7}$	1.01 $\times \;10^{-6}$
Reaction force (kgf)	1.26745	1.26878
Applied force (N)	15	15
Applied pressure (Pa)	98,066.5	98,066.5
Number of elements	63,333	97,442
Number of nodes	106,545	152,557

**Table 6:** Qualitative comparison of insulin delivery approach.

Feature	Syringe	Insulin Pen	Pump	Injection Facilitator	Proposed Applicator	References
Low cost	✓	×	×	∆	✓	[19,20,87,88]
Portability	✓	✓	∆	✓	✓	[19,21,88]
Requires technical training	High	Medium	High	Medium	Low	[22,23,25,47]
Supports correct injection	×	∆	✓	✓	✓	[31,44,45,89]
Generates skin fold	×	×	×	∆	✓	[31,45,46]
Compatible with standard syringes	✓	×	×	∆	✓	[19,20,87]
Electronic components required	×	∆	✓	×	×	[21,88,90]

✓ present, ∆ partially support, × not supported.

## Data Availability

Dataset available on request from the authors.

## References

[1] Pan American Health Organization/World Health Organization (2022). Prevalence of Diabetes and Diabetes Treatment Coverage—ENLACE. https://www.paho.org/en/enlace/prevalence-diabetes-and-diabetes-treatment-coverage.

[2] Pan American Health Organization (2025). Diabetes—PAHO/WHO. https://www.paho.org/en/topics/diabetes.

[3] Basto-Abreu A.C., López-Olmedo N., Rojas-Martínez R., Aguilar-Salinas C.A., De la Cruz-Góngora V.V., Rivera-Dommarco J., Shamah-Levy T., Romero-Martínez M., Barquera S., Villalpando S. (2021). Prevalence of diabetes and glycemic control in Mexico: National results from 2018 and 2020. Salud Pública Méx..

[4] World Health Organization (2016). Global Report on Diabetes.

[5] International Diabetes Federation (2025). Mexico—IDF South Central America (SACA) Region. https://idf.org/our-network/regions-and-members/south-and-central-america/members/mexico/.

[6] Basto-Abreu A., López-Olmedo N., Rojas-Martínez R., Aguilar-Salinas C.A., Moreno-Banda G.L., Carnalla M., Rivera J.A., Romero-Martinez M., Barquera S., Barrientos-Gutiérrez T. (2023). Prevalencia de prediabetes y diabetes en México: Ensanut 2022. Salud Pública Méx..

[7] Basto-Abreu A., Barrientos-Gutiérrez T., Rojas-Martínez R., Aguilar-Salinas C.A., López-Olmedo N., De la Cruz-Góngora V., Rivera-Dommarco J., Shamah-Levy T., Romero-Martínez M., Barquera S. (2019). Prevalencia de diabetes y descontrol glucémico en México: Resultados de la Ensanut 2016. Salud Pública Méx..

[8] Barquera S., Hernández-Barrera L., Trejo B., Shamah T., Campos-Nonato I., Rivera-Dommarco J. (2020). Obesidad en México, prevalencia y tendencias en adultos. Ensanut 2018-19. Salud Pública Méx..

[9] World Health Organization (2024). Diabetes. https://www.who.int/news-room/fact-sheets/detail/diabetes.

[10] Falcetta P., Aragona M., Bertolotto A., Bianchi C., Campi F., Garofolo M., Del Prato S. (2022). Insulin discovery: A pivotal point in medical history. Metab.-Clin. Exp..

[11] Malone J.K., Anderson J.H.J., Wolpert H.A., Ilag L.L., Frank B.H., De Felippis M.R., Paavola C.D., Orr A.L., Beals J.M. (2020). Eli Lilly and Company Insulins—A Century of Innovation. Pediatr. Endocrinol. Rev..

[12] Langerman C., Forbes A., Robert G. (2024). A qualitative study of the experiences of insulin use by older people with type 2 diabetes mellitus. BMC Prim. Care.

[13] Valencia W.M., Florez H. (2014). Pharmacological treatment of diabetes in older people. Diabetes Obes. Metab..

[14] Kirkman M.S., Briscoe V.J., Clark N., Florez H., Haas L.B., Halter J.B., Huang E.S., Korytkowski M.T., Munshi M.N., Odegard P.S. (2012). Diabetes in older adults: A consensus report. J. Am. Geriatr. Soc..

[15] Abdelhafiz A.H., Rodríguez-Mañas L., Morley J.E., Sinclair A.J. (2015). Hypoglycemia in older people—A less well recognized risk factor for frailty. Aging Dis..

[16] Kesavadev J., Das A.K., Unnikrishnan R., Joshi S.R., Ramachandran A., Shamsudeen J., Krishnan G., Jothydev S., Mohan V. (2010). Use of insulin pumps in India: Suggested guidelines based on experience and cultural differences. Diabetes Technol. Ther..

[17] Garg S.K., Rewers A.H., Akturk H.K. (2018). Ever-Increasing Insulin-Requiring Patients Globally. Diabetes Technol. Ther..

[18] Knutsen P.G., Voelker C.Q., Nikkel C.C. (2015). Clinical Insights Into a New, Disposable Insulin Delivery Device. Diabetes Spectr..

[19] Joshi S.R., Kesavadev J., Saboo B., Parikh R., Chawla M., Gupta A., Bhartia M., Shankar A., Basanth A., Krishnan G. (2023). Advancements in Insulin Delivery Technology: A Journey of Evolution. Int. J. Diabetes Technol..

[20] Kesavadev J., Saboo B., Krishna M.B., Krishnan G. (2020). Evolution of Insulin Delivery Devices: From Syringes, Pens and Pumps to DIY Artificial Pancreas. Diabetes Ther..

[21] Daly A., Hovorka R. (2021). Technology in the management of type 2 diabetes: Present status and future prospects. Diabetes Obes. Metab..

[22] Steenkamp D., Eby E.L., Gulati N., Liao B. (2021). Adherence and Persistence to Insulin Therapy in People with Diabetes: Impact of Connected Insulin Pen Delivery Ecosystem. J. Diabetes Sci. Technol..

[23] Spain C.V., Wright J.J., Hahn R.M., Wivel A., Martin A.A. (2016). Self-reported Barriers to Adherence and Persistence to Treatment with Injectable Medications for Type 2 Diabetes. Clin. Ther..

[24] Mathew B.K., De Roza J.G., Liu C., Goh L.J., Ooi C.W., Chen E., Tang W.E. (2022). Which Aspect of Patient–Provider Relationship Affects Acceptance and Adherence of Insulin Therapy in Type 2 Diabetes Mellitus? A Qualitative Study in Primary Care. Diabetes Metab. Syndr. Obes. Targets Ther..

[25] Consoli A., Formoso G. (2023). Patient perceptions of insulin therapy in diabetes self-management with insulin injection devices. Acta Diabetol..

[26] Trandafir L.M., Moisa S.M., Vlaiculescu M.V., Butnariu L.I., Boca L.O., Constantin M.M.L., Lupu P.M., Brinza C., Temneanu O.R., Burlacu A. (2022). Insulin Pump Therapy Efficacy and Key Factors Influencing Adherence in Pediatric Population—A Narrative Review. Medicina.

[27] Maltoni G., Zioutas M., Zucchini S., Pession A. (2018). Using an injection port helps improve metabolic control and compliance to a strict basal-bolus regimen in children and adolescents with type 1 diabetes. J. Diabetes.

[28] Al Hayek A.A., Robert A.A., Al Dawish M.A. (2021). Efficacy of i-Port Advance system on patients satisfaction and glycemic control among patients with type 1 diabetes in Saudi Arabia. Diabetes Metab. Syndr. Clin. Res. Rev..

[29] Dostal P., Taubel J., Lorch U., Aggarwal V., York T. (2023). The Reliability of Auto-Injectors in Clinical Use: A Systematic Review. Cureus.

[30] Pineo R. (2026). Mexico’s Failing Healthcare System: The Past and Present of Healthcare and Public Health in Mexico. J. Dev. Soc..

[31] Heinemann L., Nguyen T., Bailey T.S., Hassoun A., Kulzer B., Oliveria T., Reznik Y., de Valk H.W., Mader J.K. (2021). Needle Technology for Insulin Administration: A Century of Innovation. J. Diabetes Sci. Technol..

[32] Wang J.K., Pamnani R.D., Capasso R., Chang R.T. (2018). An Extended Hackathon Model for Collaborative Education in Medical Innovation. J. Med. Syst..

[33] Rodriguez N.M., Burleson G., Linnes J.C., Sienko K.H. (2023). Thinking Beyond the Device: An Overview of Human- and Equity-Centered Approaches for Health Technology Design. Annu. Rev. Biomed. Eng..

[34] Nakao K., Umezu M., Iwasaki K. (2022). Biodesign program introduction in Japan: Promotion of entrepreneurship and viewpoints of education on medical technology innovation. J. Artif. Organs.

[35] Augustin D.A., Yock C.A., Wall J., Lucian L., Krummel T., Pietzsch J.B., Azagury D.E. (2020). Stanford’s Biodesign Innovation program: Teaching opportunities for value-driven innovation in surgery. Surgery.

[36] Wei X., Wang Y., Liu Y., Ji K., Li K., Wang J., Gu Z. (2024). Biomimetic design strategies for biomedical applications. Matter.

[37] Duran M.M., Moro G., Zhang Y., Islam A. (2023). 3D Printing of Silicone and Polyurethane Elastomers for Medical Device Application: A Review. Adv. Ind. Manuf. Eng..

[38] Miranda I., Souza A., Sousa P., Ribeiro J., Castanheira E.M.S., Lima R., Minas G. (2022). Properties and Applications of PDMS for Biomedical Engineering: A Review. J. Funct. Biomater..

[39] Qaiser N., Suryaprabha T., Vetrano F., Hwang B., Elatab N. (2026). Mechanics and Bio-Interface Engineering in Flexible Biosensors for Continuous Health Monitoring. npj Flex. Electron..

[40] Boufidis D., Garg R., Angelopoulos E., Cullen D.K., Vitale F. (2025). Bio-inspired electronics: Soft, biohybrid, and “living” neural interfaces. Nat. Commun..

[41] Büscher T.H., Gorb S.N. (2026). Adhesion and Friction in Biological and Bioinspired Systems. Biomimetics.

[42] Callens S.J.P., Fan D., van Hengel I.A.J., Minneboo M., Díaz-Payno I.A.J., Stevens M.M., Fratila-Apachitei L.E., Zadpoor A.A. (2023). Emergent collective organization of bone cells in complex curvature fields. Nat. Commun..

[43] Thanigaiarasu P., Timiri Shanmugam P.S., Chokkalingam L., Bakthavachalam P. (2020). 3—Biomimetics in the design of medical devices. Trends in Development of Medical Devices.

[44] Frid A.H., Kreugel G., Grassi G., Halimi S., Hicks D., Hirsch L.J., Smith M.J., Wellhoener R., Bode B.W., Hirsch I.B. (2016). New Insulin Delivery Recommendations. Mayo Clin. Proc..

[45] Kalra S., Pathan F., Kshanti I.A.M., Bay N.Q., Nagase T., Oliveria T., Bajpai S. (2023). Optimising Insulin Injection Techniques to Improve Diabetes Outcomes. Diabetes Ther..

[46] Gibney M.A., Arce C.H., Byron K.J., Hirsch L.J. (2010). Skin and subcutaneous adipose layer thickness in adults with diabetes at sites used for insulin injections: Implications for needle length recommendations. Curr. Med. Res. Opin..

[47] Boye K.S., Jordan J.B., Malik R.E., Currie B.M., Matza L.S. (2021). Patient Perceptions of and Preferences Between Characteristics of Injectable Diabetes Treatments. Diabetes Ther..

[48] Companion Medical (2023). InPen System: Instructions for Use. https://www.medtronicdiabetes.com/sites/default/files/library/download-library/user-guides/InPen-user-guide.pdf.

[49] Adolfsson P., Hartvig N.V., Kaas A., Møller J.B., Hellman J. (2020). Increased Time in Range and Fewer Missed Bolus Injections After Introduction of a Smart Connected Insulin Pen. Diabetes Technol. Ther..

[50] Chien A., Thanasekaran S., Gaetano A., Im G., Wherry K., MacLeod J., Vigersky R.A. (2023). Potential cost savings in the United States from a reduction in sensor-detected severe hypoglycemia among users of the InPen smart insulin pen system. J. Manag. Care Spec. Pharm..

[51] Innovation Zed (2021). Injection Pen Technology. https://innovationzed.com.

[52] Bigfoot Biomedical (2023). Bigfoot Unity Diabetes Management System. https://www.bigfootbiomedical.com/bigfoot-unity.

[53] Baliga B.S., Tillman J.B., Olson B., Vaughan S., Sheikh F.N., Malone J.K. (2023). First Real-World Experience with Bigfoot Unity: A 6-Month Retrospective Analysis. Clin. Diabetes.

[54] Eli Lilly (2023). Instructions for Use (Tempo Smart Button). https://pi.lilly.com/us/tempo-smart-button-ug.pdf.

[55] Novo Nordisk (2023). NovoPen 6 User Guide. https://www.novonordisk.com/content/dam/nncorp/global/en/our-products/pdf/instructions-for-use/novopen-6/NovoPen6-ES.pdf.

[56] Novo Nordisk (2023). NovoPen Echo Plus User Guide. https://www.novonordisk.com/content/dam/nncorp/global/en/our-products/pdf/instructions-for-use/novopen-echo-plus/Novopen-echo-plus-ES.pdf.

[57] Ypsomed (2022). YpsoMate—Quick Guide. https://www.ypsomed.com/en/products/autoinjectors/ypsomate.

[58] Mannucci E., Pintaudi B., Lunati M.E., Fiorina P. (2025). Needle Characteristics and the Insulin Injection Experience in Patients with Diabetes. Acta Diabetol..

[59] Jordan M.A., Choksi D., Lombard K., Patton L.R. (2021). Development of Guidelines for Accurate Measurement of Small Volume Parenteral Products Using Syringes. Hosp. Pharm..

[60] Lim D., Song M., Kim M., Park H.K., Kim D.W., Pang C. (2025). Bioinspired Suction-Driven Strategies with Nanoscale Skin-Controllable Adhesive Architectures for Efficient Liquid Formulated Transdermal Patches. ACS Nano.

[61] Hamidi M.N., Abdullah J., Mahmud A.S., Namazi H. (2026). Effect of blend ratios and printing parameters on PLA/TPU shape memory polymer performance. Polym. Test..

[62] Ramos I., Gonçalves M., Gonçalves I.M., Carvalho V., Fernandes E., Lima R., Pinho D. (2025). PDMS Surface Wettability Modification and Its Applications: A Systematic Review. J. Mol. Liq..

[63] Razavi M., Primavera R., Vykunta A., Thakor A.S. (2021). Silicone-Based Bioscaffolds for Cellular Therapies. Mater. Sci. Eng. C Mater. Biol. Appl..

[64] Gradinariu A.I., Racles C., Stoica I., Stelea C.G., Simionescu A.M.A., Jehac A.E., Costan V.V. (2024). Silicones for Maxillofacial Prostheses and Their Modifications in Service. Materials.

[65] Sounouvou H.T., Lechanteur A., Piel G., Evrard B. (2022). Silicones in Dermatological Topical Drug Formulation: Overview and Advances. Int. J. Pharm..

[66] Zare M., Ghomi E.R., Venkatraman P.D., Ramakrishna S. (2021). 3D Printing of Polymeric Materials: Applications in Biomedical Engineering. J. Appl. Polym. Sci..

[67] Chruściel J.J. (2025). Most Important Biomedical and Pharmaceutical Applications of Silicones. Materials.

[68] Barmouz M., Azarhoushang B., Kintzel W., Bucher V. (2025). Long-Term Impact of Sterilization Cycles on the Surface and Mechanical Integrity of Medical-Grade Silicone. J. Manuf. Mater. Process..

[69] Sim O., Jeong B.C., Lee C. (2025). Verification, Validation, and Uncertainty Quantification of Finite Element Analysis Results for Pedicle Screw Assemblies under ASTM F1717 Flexion and Extension Testing. Front. Bioeng. Biotechnol..

[70] Nering K., Nering K. (2023). A Low-Stress Method for Determining Static and Dynamic Material Parameters for Vibration Isolation with the Use of VMQ Silicone. Materials.

[71] Pal S., Bhattacharyya A. (2025). Measurement of axial and shear mechanical response of PDMS elastomers and determination of Poisson’s ratio using digital image correlation. Polym. Test..

[72] Yoo S., Lv Z., Fadell N., Yoo J.Y., Oh S., Ha K.H., Moritz W.M., Cha J., Wu H., Park J. (2026). A Wireless, Skin-Integrated System for Continuous Pressure Distribution Monitoring to Prevent Ulcers across Various Healthcare Environments. npj Flex. Electron..

[73] Riddle M., MacDermid J., Robinson S., Szekeres M., Ferreira L., Lalone E. (2020). Evaluation of individual finger forces during activities of daily living in healthy individuals and those with hand arthritis. J. Hand Ther..

[74] McNulty A.K., Wilkes R.P., Marchand B., Ingram S., Mann S., Sieracki J. (2025). Finite Element and Preclinical Analysis of Tissue Response to Negative Pressure Wound Therapy with a Felted Foam Containing 10 mm Through Holes. Front. Bioeng. Biotechnol..

[75] Cai F., Jiang X., Hou X., Wang D., Wang Y., Deng H., Guo H., Wang H., Li X. (2021). Application of Infrared Thermography in the Early Warning of Pressure Injury: A Prospective Observational Study. J. Clin. Nurs..

[76] Baron M.V., Hernandes Martins P.R., Brandenburg C., Koepp J., Reinheimer I.C., Dos Santos A.C., Dos Santos M.P., Mantilla Santamaria A.F., Miliou T., da Costa B.E.P. (2023). Accuracy of Thermographic Imaging in the Early Detection of Pressure Injury: A Systematic Review. Adv. Ski. Wound Care.

[77] Jordan K., John G.T., Chung A., Asare-Baiden M., Hertzberg V.S., Ho J.C., Sonenblum S.E. (2025). Impact of Skin Tone and Cupping on Erythema and Thermal Imaging Measurements. Sci. Rep..

[78] Gomes V.M.d.S.A., Tenório N., Silva A.R.C.d., Oliveira L.R.P., Silva A.C.S.d., Maia J.N., Brioschi M.L., Dantas D. (2024). Reproducibility of Thermography for Measuring Skin Temperature of Upper Limbs in Breast Cancer Survivors. Biomedicines.

[79] Zoboli L., Luppino F., Bianchi D., Nannei A., Lazzarotti L., Centola M., Gizzi A. (2024). Multi-Field Modeling and Computational Optimization of a Subcutaneous Insulin Injection Port. Mater. Des..

[80] Dassault Systèmes SolidWorks Corporation (2011). SolidWorks Flow Simulation: Student Workbook. https://www.solidworks.com/sw/docs/flow_sim_studentwb_2011_deu.pdf.

[81] Sparre T., Hammershøy L., Steensgaard D.B., Sturis J., Vikkelsøe P., Azzarello A. (2023). Factors Affecting Performance of Insulin Pen Injector Technology: A Narrative Review. J. Diabetes Sci. Technol..

[82] Young E., Gutschmidt S., Chase J.G. (2025). Measuring the Mechanical Properties of Insulin: A Potential Solution to Overcoming the Challenges of Real-Time, Point-of-Care Insulin Sensing. J. Diabetes Sci. Technol..

[83] Deosarkar S.D., Pawar H.N., Dudhate R.V., Bhosale B.R., Arsule A.D., Kalyankar T.M. (2025). Analysis of Molecular Interactions in Glucose-Aqueous-Human Mixtard Insulin/Metformin Solutions by Volumetric, Acoustic Properties. Chem. Thermodyn. Therm. Anal..

[84] Yi L., Shuai T., Tian X., Zeng Z., Ma L., Song G.M. (2016). The effect of subcutaneous injection duration on patients receiving low-molecular-weight heparin: Evidence from a systematic review. Int. J. Nurs. Sci..

[85] Sadafi H., Monshi Tousi N., De Backer W., De Backer J. (2024). Validation of Computational Fluid Dynamics Models for Airway Deposition with SPECT Data of the Same Population. Sci. Rep..

[86] Seok M., Choi Y., Cho Y.H. (2023). Reusable and Porous Skin Patches with Thermopneumatic Adhesion Control Capability and High Water Vapor Permeability. ACS Appl. Mater. Interfaces.

[87] Kamrul-Hasan A.B.M., Hannan M.A., Alam M.S., Rahman M.M., Asaduzzaman M., Mustari M., Paul A.K., Kabir M.L., Chowdhury S.R., Talukder S.K. (2023). Comparison of Simplicity, Convenience, Safety, and Cost-Effectiveness Between Use of Insulin Pen Devices and Disposable Plastic Syringes by Patients with Type 2 Diabetes Mellitus: A Cross-Sectional Study from Bangladesh. BMC Endocr. Disord..

[88] Lingen K., Pikounis T., Bellini N., Isaacs D. (2023). Advantages and Disadvantages of Connected Insulin Pens in Diabetes Management. Endocr. Connect..

[89] Gorska-Ciebiada M., Masierek M., Ciebiada M. (2020). Improved Insulin Injection Technique, Treatment Satisfaction and Glycemic Control: Results From a Large Cohort Education Study. J. Clin. Transl. Endocrinol..

[90] Davis G.M., Hughes M.S., Brown S.A., Sibayan J., Perez-Guzman M.C., Stumpf M., Thompson Z., Basina M., Patel R.M., Hester J. (2023). Automated Insulin Delivery with Remote Real-Time Continuous Glucose Monitoring for Hospitalized Patients with Diabetes: A Multicenter, Single-Arm, Feasibility Trial. Diabetes Technol. Ther..

